# Computational Multi-Scale Modeling of Drug Delivery into an Anti-Angiogenic Therapy-Treated Tumor

**DOI:** 10.3390/cancers15225464

**Published:** 2023-11-17

**Authors:** Mahya Mohammadi, Mostafa Sefidgar, Cyrus Aghanajafi, Mohammad Kohandel, M. Soltani

**Affiliations:** 1 Department of Mechanical Engineering, K. N. Toosi University of Technology, Tehran 19919-43344, Iran; mahya.mohammadi@email.kntu.ac.ir (M.M.); aghanajafi@kntu.ac.ir (C.A.); 2 Department of Mechanical Engineering, Pardis Branch, Islamic Azad University, Pardis 16581-74583, Iran; msefidgar@pardisiau.ac.ir; 3 Department of Applied Mathematics, University of Waterloo, Waterloo, ON N2L 3G1, Canada; kohandel@uwaterloo.ca; 4 Department of Electrical and Computer Engineering, University of Waterloo, Waterloo, ON N2L 3G1, Canada; 5 Centre for Biotechnology and Bioengineering (CBB), University of Waterloo, Waterloo, ON N2L 3G1, Canada; 6 Department of Integrative Oncology, BC Cancer Research Institute, Vancouver, BC V5Z 1L3, Canada; 7 Centre for Sustainable Business, International Business University, Toronto, ON M5S 2V1, Canada

**Keywords:** drug delivery, anti-angiogenesis, intravascular blood flow, interstitial fluid flow, dynamic model, heterogeneous microvascular network, angiostatin, convection-diffusion equation, vascular normalization

## Abstract

**Simple Summary:**

The investigation of chemotherapy combined with anti-angiogenesis has garnered significant attention from researchers. The aim of this study is to provide a numerical model, the first of its kind, considering more realistic phenomena in simulating drug delivery into a solid tumor with a remodeled dynamic microvascular network affected by the anti-angiogenic agent, angiostatin. This research aims to open a new horizon in understanding the efficiency of combination therapy involving anti-angiogenesis and chemotherapy. Results show that, for improving drug delivery with the aid of anti-angiogenesis, the uniformity of micro-vessel distribution, accompanied by the modification in drug exposure schedule caused by the alterations in transport properties induced by vascular normalization, is more effective than the suppression of the microvasculature. Therefore, it can be concluded that the 39% enhancement in uniformity of drug delivery in R = 0.2 cm is a result of the well-proportioned distribution of the capillary network in the second approach of anti-angiogenic therapy.

**Abstract:**

The present study develops a numerical model, which is the most complex one, in comparison to previous research to investigate drug delivery accompanied by the anti-angiogenesis effect. This paper simulates intravascular blood flow and interstitial fluid flow using a dynamic model. The model accounts for the non-Newtonian behavior of blood and incorporates the adaptation of the diameter of a heterogeneous microvascular network derived from modeling the evolution of endothelial cells toward a circular tumor sprouting from two-parent vessels, with and without imposing the inhibitory effect of angiostatin on a modified discrete angiogenesis model. The average solute exposure and its uniformity in solid tumors of different sizes are studied by numerically solving the convection-diffusion equation. Three different methodologies are considered for simulating anti-angiogenesis: modifying the capillary network, updating the transport properties, and considering both microvasculature and transport properties modifications. It is shown that anti-angiogenic therapy decreases drug wash-out in the periphery of the tumor. Results show the decisive role of microvascular structure, particularly its distribution, and interstitial transport properties modifications induced via vascular normalization on the quality of drug delivery, such that it is improved by 39% in uniformity by the second approach in R = 0.2 cm.

## 1. Introduction

Investigation of different methodologies to improve the quality of drug delivery and the effectiveness of chemotherapy is of great interest among researchers [1,2]. Abnormally tortuous tumor microvasculature generated via the angiogenesis process is one of the main reasons for unsuccessful drug delivery [3,4]. Therefore, anti-angiogenesis, an adjuvant treatment strategy [5], needs to be studied, especially by using mathematical modeling [6]. The concept of anti-angiogenesis was introduced by Folkman [7] regarding the prevention of capillaries’ sprouting into the tumor site. It has been reported in clinical studies [8,9,10] and review papers [11,12,13] that anti-angiogenic drug administration improves chemotherapeutic drug delivery, its efficiency, and its penetration depth.

The modeling of solid tumors involves multiple spatial and temporal scales of complexity [14,15]. The formation of a tumor-induced capillary network in nanometers, intravascular blood flow in the micrometer dimension, blood flow distribution in the capillary network in millimeters, and fluid flow and solute transport in tumor and normal tissues on the scale of centimeters are all examples of multi-scale modeling of cancer-related studies [16].

Mathematical and computational studies have made great strides in cancer modeling to provide qualitative and quantitative comprehension of the complex dynamics of cancer. Hadjicharalambous et al. [17] conducted a review of in silico studies that assessed tumor perfusion, angiogenesis, drug delivery, and investigations leveraging clinical data. Jain and his colleagues [18,19,20] conducted basic studies on drug delivery into solid tumors. They considered tumor tissue as a porous medium and introduced high interstitial fluid pressure (IFP) as one of the main barriers to effective drug delivery. In 2007, Jain et al. [21] studied vascular normalization by modeling the interstitial fluid flow in a macroscopic model. They showed that IFP decreased after vascular normalization. This model was improved to consider solute transport analysis in a single tumor nodule [22] and a non-homogeneous macroscopic model [23,24]. Time course of perfusion was introduced as a controlling factor of normalization efficiency, which depends on tumor size, normalization intensity, and concurrent therapeutic agents [23].

Mathematical modeling of angiogenesis should be considered for extracting the capillary network to develop a microscopic analysis. Anderson and Chaplain [25,26] developed a mathematical study on continuous and discrete models of angiogenesis, which is the base of different study [27,28,29]. In our recent study [30,31], the mathematical model of angiogenesis was modified to consider the effect of proliferation and death of endothelial cells and matrix-degrading enzymes. The morphology of a tumor-induced microvascular network with two parent vessels was simulated under the inhibitory effect of an anti-angiogenic agent, angiostatin.

Though the macroscopic view of the tumor microenvironment could provide a qualitative description of different phenomena of cancer, considering the microscopic capillary network of the tumor is an important factor in achieving a more realistic illustration. Stéphanou et al. [32] and McDougall et al. [33] investigated tumor-induced angiogenesis via a mathematical model that simultaneously simulated blood flow and dynamic capillary network progression by considering the non-Newtonian blood behavior and non-uniform distribution of the hematocrit in the bifurcations. The objective of their research was to develop a model to make tumor-induced angiogenesis more precise. Accordingly, they did not consider transvascular flow and interstitial fluid flow. Alamer and Xu [34] studied the effect of microvasculature on interstitial fluid flow and investigated vascular normalization via capillary pruning. Their model had the limitation of not considering non-Newtonian blood behavior and the adaptation of micro-vessel diameter in response to transmural pressure and metabolic stimuli. Soltani and Chen [35] investigated interstitial fluid flow distribution in relation to blood flow in a dynamic tumor-induced microvasculature from one parent vessel by considering the non-continuous behavior of blood. Sefidgar et al. [16] further developed the model of Soltani and Chen [35] to include solute transport analysis to reflect drug distribution in the tumor site. Wu et al. [27] used a mathematical model of angiogenesis, which produces capillary networks originating from two distinct vessels of arteriole and venule, to study blood flow and interstitial flow distribution by decreasing the capillary network’s density to mimic the anti-angiogenic effect. In another study by this group [36], the anti-angiogenic effect of angiostatin and endostatin in combination with intravascular and interstitial flow is considered. The blood vessels in this study were assumed to be dynamic based on the compliance method of Netti in the tumor tissue. In another study [37], the anti-angiogenic effect of angiostatin on interstitial fluid flow behavior was investigated in a rigid capillary network without considering the non-continuous behavior of blood. 

Ozturk et al. [38] investigated the effect of vascular normalization on liposome delivery into a homogeneous solid tumor based on a study by Jain et al. [21] They found that the efficiency of normalization in improving liposome delivery is a function of tumor size. Stylianopoulos and Jain [39] investigated the effect of vascular normalization by assuming a decrease in micro-vessels’ diameter and their pruning. They concluded that normalization is more effective in microvasculature with more permeability and less compressibility characteristics.

Steuperaert et al. [40] investigated intraperitoneal drug delivery using a macroscopic model based on an actual image extracted via magnetic resonance imaging. They also took into account interstitial transport properties, considering a non-uniform distribution. Image processing techniques were used to develop a macroscopic model [41] of a brain tumor to investigate the effect of administration of bevacizumab on drug delivery. Sweeney et al. [42] studied anti-angiogenesis by applying the modifications to transport properties in solid tumors with a microvasculature based on the real image.

To develop a comprehensive numerical microscopic model that simulates the effect of anti-angiogenic adjuvant therapy on the quality of drug delivery, one should consider multi-physics in a multi-scale context. This model should describe (a) the generation of tumor-induced angiogenesis under the inhibitory effect of the anti-angiogenic agent, angiostatin in this study; (b) intravascular blood flow in connection with interstitial fluid flow in a dynamic microvasculature, taking into account the non-Newtonian behavior of blood and adapting the micro-vessels’ diameter in response to hemodynamic and metabolic stimuli; and (c) spatiotemporal solute transport into the tumor tissue. All of these aspects are addressed in the present paper for the first time. Consideration of different tumor sizes and vascular normalization approaches are other contributions of this paper. Three approaches are observed for investigating anti-angiogenic therapy. In the first approach, the microvasculature is updated under the influence of an anti-angiogenic agent without considering modifications in transport properties. In the second approach, modifications in transport properties experimentally induced via anti-angiogenesis are considered. The third approach applies the model by combining the former two.

## 2. Materials and Methods

### 2.1. Computational Model Geometry

**Figure 1:**
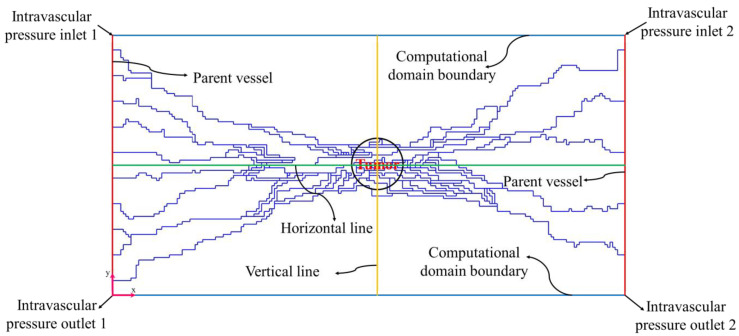
Schematic view of the computational domain, coordinate origin, parent vessels, cut lines, and boundaries.

In this study, a circular tumor (with different radius sizes of R=0.8 cm, R=0.6 cm, R=0.4 cm, and R=0.2 cm) surrounded by normal tissue (a rectangular domain of 2×4 cm) is considered. A parent vessel with ten sprouts is defined on the left side of the tumor, and one parent vessel with five sprouts is located on the right side of the tumor. Figure 1 shows an illustration of the computational field, which is rendered in a two-dimensional domain.

### 2.2. Governing Equations

In this multi-scale study, angiogenesis and network formation under the influence of an anti-angiogenic agent, blood flow distribution in microvascular networks, and interstitial fluid flow and solute transport in tumor and normal tissues are mathematically modeled. The following sections describe the equations that govern the physics of each phenomenon.

#### 2.2.1. Angiogenesis and Anti-Angiogenesis

In this study, a tumor-induced microvascular network was simulated via a probabilistic model of reinforced random walk, which was developed based on the discretized non-dimensional governing equations of evolution of endothelial cells (ECs) in a discrete model. Anderson and Chaplain [25,26] proposed this model by initially considering three main mechanisms, i.e., random motility, chemotaxis, and haptotaxis, that control ECs’ migration to the tumor site. This model was further developed in our recent study [30] to consider the sprouting of two-parent vessels toward a circular tumor. Moreover, the proliferation and death of ECs and the effect of matrix-degrading enzyme was considered. For simulating the response of tumor-induced angiogenesis to the anti-angiogenic agent, the model was modified to consider the effect of angiostatin. Then, different microvascular networks were extracted in different tumor sizes. More details were provided in [30].

#### 2.2.2. Intravascular Blood Flow

The model utilized in this study to simulate the apparent blood viscosity is based on the work of Pries et al. [43,44]. They incorporated data from 18 studies on human blood and integrated them with a parametric representation of apparent blood viscosity in relation to plasma viscosity in order to define the apparent viscosity. The empirical equation formulated by Pries and Secomb [45] was employed to characterize the distribution of blood hematocrit in bifurcations. The dynamic microvascular model developed by Pries and colleagues [46,47,48] was chosen to model the structural adaptation of micro-vessels. In terms of accuracy, the Pries model was validated by comparison to the experimental data. This model considers determinative factors in microcirculatory behavior, i.e., hematocrit, viscosity, and micro-vessel diameter. Regarding its applicability, the Pries model is considered to be quite versatile and is frequently employed in studies related to developmental remodeling. Many researchers regard it as the fundamental model for vascular remodeling [49].

**Figure 2:**
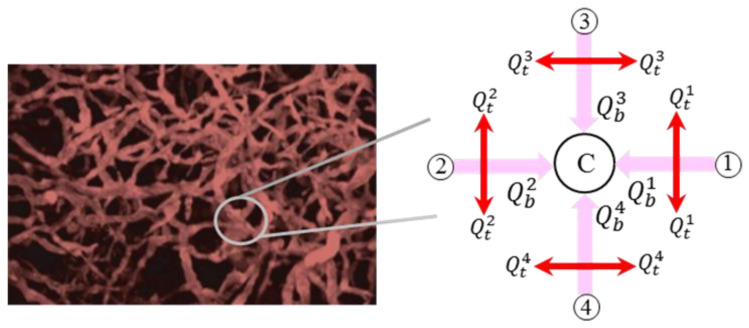
Schematic view of the flow in each node of the microvascular network.

A capillary network is analogous to an electronic circuit. Similar analyses for solving electronic circuits in capillary networks can be used if the pressure and the volumetric flow rate are set as equal to the electric potential and current, respectively. A pressure-dependent linear equation system that allows for the calculation of pressure and flow is obtained by considering the volumetric flow rate conservation at each node of the network. The conservation of mass for each node, such as c, which is shown in Figure 2, can be written as [16,35];

(1) $$\sum_{k=1}^{N}Q_{c}^{k}\beta_{k}=0$$

where the index k represents the adjacent nodes and N is the number of adjacent nodes, which is 4 for a fully connected network in this manuscript. $\beta_{k}$ is zero or one, which indicates the presence or absence of a connection between point c and its adjacent point k. $Q_{c}^{k}$ is the net flow for each capillary, expressed in Equation (2).

(2) $$Q_{c}^{k}=Q_{B,c}^{k}-Q_{t,c}^{k}$$

**Figure 3:**
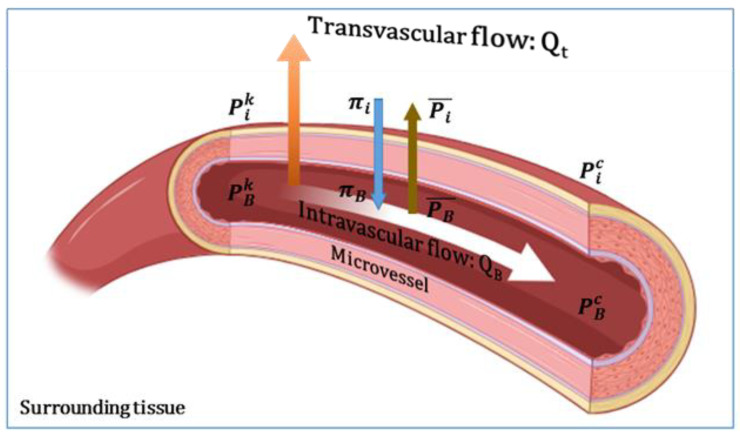
Schematic illustration of the intravascular and extravascular flow. Redrawn from [35].

$Q_{B,c}^{k}$ and $Q_{t,c}^{k}$ are the intravascular flow in each capillary and the transvascular flow from or to the capillary wall, respectively, which are also shown in Figure 3.

Since the Reynolds number of blood flow in the capillaries is less than 1 (laminar regime), Poiseuille’s law can be used as follows [35]:

(3) $$Q_{B,c}^{k}=\frac{\pi }{128}\frac{\Delta PD^{4}}{L\mu }$$

where $\Delta P=P_{B}^{c}-P_{B}^{k}$, D, L, and μ show the driving pressure, the diameter of the capillary, the length of the capillary, and the blood viscosity, respectively.

The transvascular flow rate through the capillary wall is expressed by Starling’s model as follows [50]:

(4) $$Q_{t,c}^{k}=\pi DLL_{p}({\bar{P}}_{B}-{\bar{P}}_{i}-\sigma_{s}(\pi_{B}-\pi_{i}))$$

${\bar{P}}_{B}=\frac{P_{B}^{c}+P_{B}^{k}}{2}$ is considered as the average intravascular pressure between node c and adjacent node k [35]. ${\bar{P}}_{i}$ is defined with the equation of ${\bar{P}}_{i}=\frac{P_{i}^{c}+P_{i}^{k}}{2}$ and represents the average IFP between node c and adjacent node k [35]. $L_{p}$ is the hydraulic conductivity of the microvascular wall. $\sigma_{s}$ shows the average osmotic reflection coefficient for plasma proteins. $\pi_{B}$ and $\pi_{i}$ demonstrate the osmotic pressure of the plasma and the osmotic pressure of the interstitial fluid, respectively [18].

Combining Equations (1)–(4) results in:

(5) $$\sum_{k=1}^{N}(\frac{\pi }{128}\frac{\Delta PD^{4}}{L\mu }-\pi DLL_{p}({\bar{P}}_{B}-{\bar{P}}_{i}-\sigma_{s}(\pi_{B}-\pi_{i})))\beta_{k}=0$$

The transvascular flow rate depends on the intravascular and interstitial pressures. The intravascular pressure is calculated by solving the mass conservation equation at each network node (Equation (1)). Solving the equation’s governing fluid flow in the porous medium yields the IFP around the capillary network. Equation (4) is a bridge between the blood flow in the microvascular network and fluid flow in the surrounding tissue.

##### Blood Viscosity

Blood is a non-Newtonian fluid with four main components, namely, plasma, red blood cells, white blood cells, and platelets. To use Poiseuille’s law, which is applicable to Newtonian fluids, it is necessary to define the apparent blood viscosity. Blood suspension characteristics have a significant effect on the dynamics of blood flow in capillaries. The finite size of these suspended elements in capillaries causes a few important phenomena, such as non-continuum behavior, variation of blood apparent viscosity with micro-vessel diameter, and non-uniform distribution of hematocrit (volume percentage of red blood cells in the blood) between branches of diverging microvascular bifurcations [16]. Pries et al. [43,44] established the following empirical relationship for apparent blood viscosity as a function of micro-vessel diameter and hematocrit for a range of micro-vessel radius (from 2 μm to 300 μm):

(6) $$\mu_{app}=\mu_{plasma}.\mu_{\mathrm{rel}}$$

in which $\mu_{plasma}$ shows the plasma viscosity with a constant value of $1.2\times 10^{-3}[Pa.s]$. $\mu_{\mathrm{rel}}$ is the relative apparent viscosity, which is defined as follows:

(7) $$\mu_{\mathrm{rel}}=[1+(\mu_{45}-1)\frac{{\left(1-H\right)}^{C}}{{\left(1-0.45\right)}^{C}-1}{\left(\frac{D}{D-1.1}\right)}^{2}]{\left(\frac{D}{D-1.1}\right)}^{2}$$

$\mu_{45}$ denotes the relative apparent viscosity for a fixed value of hematocrit (H=0.45). D is the micro-vessel diameter. C is defined with the following function:

(8) $$C=(0.8+e^{-0.075D})(-1+\frac{1}{1+10^{-11}D^{12}})+\frac{1}{1+10^{-11}D^{12}}$$

##### Blood Hematocrit 

Microvascular blood flow includes both cells and plasma, which is what is known as a two-phase flow. A hematocrit of 100% can be found in one daughter vessel and none in the other, as the component distribution is not proportional to the plasma distribution at the branches [51]. It is found that the blood hematocrit distribution in bifurcations of the microvascular network varies based on the study by Pries and Secomb [45]. The fractional flow of red blood cells into one daughter branch ($\frac{H_{2}}{H_{1}}$) is determined using the fractional blood flow ($\frac{Q_{2}}{Q_{1}}$) as mentioned in [45]:

(9) $$\left\{ \begin{array}{ll} \frac{H_{2}}{H_{1}}=0\; & \mathrm{if}\;\frac{Q_{2}}{Q_{1}}\leq X_{0}\\ \mathrm{logit}\frac{H_{2}}{H_{1}}=A+B\mathrm{logit}[(\frac{Q_{2}}{Q_{1}}-X_{0})/(1-2X_{0})] & \mathrm{if}\;X_{0}<\frac{Q_{2}}{Q_{1}}<1-X_{0}\\ \frac{H_{2}}{H_{1}}=1\; & \mathrm{if}\;1-X_{0}\leq \frac{Q_{2}}{Q_{1}} \end{array}\right.\;(\mathrm{logit}x=\ln(\frac{x}{1-x}))\;\;\;\;\;$$

where A, B, $X_{0}$ are phase separation characteristics and defined as follows:

(10) $$\begin{array}{l} A=-13.29(\frac{\left({\left(\frac{D_{3}}{D_{2}}\right)}^{2}-1\right)}{\left({\left(\frac{D_{3}}{D_{2}}\right)}^{2}+1\right)})\frac{\left(1-H_{1}\right)}{D_{1}}\\ B=1+\frac{6.98(1-H_{1})}{D_{1}}\\ X_{0}=\frac{0.964(1-H_{1})}{D_{1}} \end{array}$$

**Figure 4:**
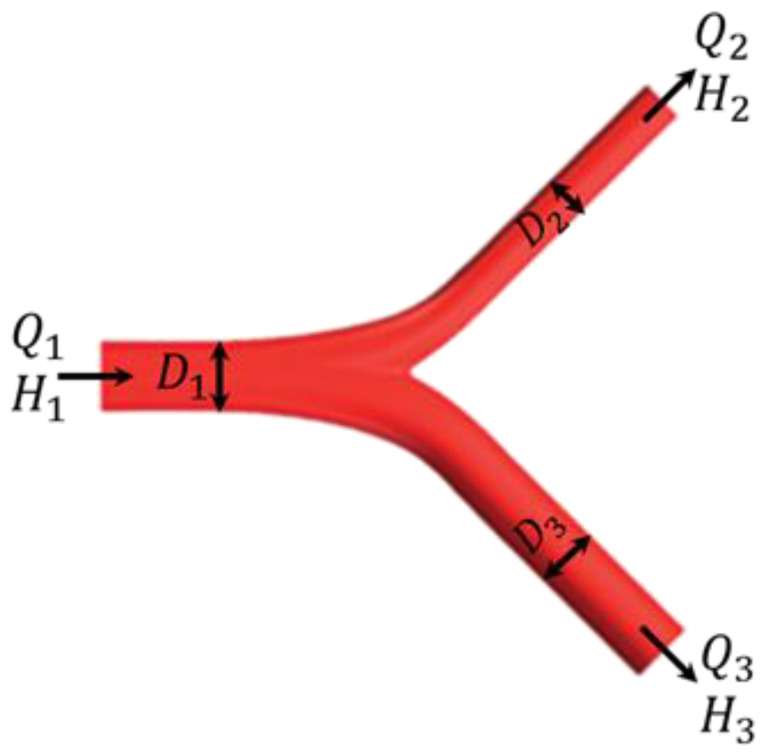
Schematic view of blood flow bifurcation. Redrawn from [16].

$D_{1}$, $D_{2}$, $D_{3}$, $H_{1}$, $H_{2}$, and $H_{3}$ are shown in Figure 4.

##### Vessel Diameter Adaptation 

The vascular structure in the human body is viscoelastic. In response to a force exerted upon the vessel wall, dilation occurs, and when the force is released, the vessel tends to shrink. Vascular dilation or contraction occurs as a result of exerted wall shear stress, intravascular pressure, and metabolic mechanisms related to the blood hematocrit [16,35]. The change in diameter (∆D) for a time step (Δt) for each segment in the microvascular network is assumed to be proportional to the stimulus term ($S_{tot}$), vessel initial diameter (D), and the time step [46,47,48]:

(11) $$\Delta D=S_{tot}D\Delta t,\;\;\;\;\;\;\;S_{tot}=S_{h}+S_{m}-k_{s}$$

in which $S_{h}$ and $S_{m}$ show the hemodynamic and metabolic stimuli, respectively. The following equations are used to define $S_{h}$ and $S_{m}$ [46,47]:

(12) $$S_{h}=\log_{10}(\tau_{w}+\tau_{\mathrm{ref}})-k_{p}\log_{10}\tau_{e}$$

(13) $$S_{m}=k_{m}\log_{10}(\frac{Q_{\mathrm{ref}}}{Q_{B}H}+1)$$

where $\tau_{w}$ shows the wall shear stress in a capillary with the equation of $\frac{32Q_{B}\mu_{app}}{\pi D^{3}}$. $\tau_{\mathrm{ref}}$ is a positive constant value (0.103  [Pa]) which is considered when the wall shear stress has a small value to avoid singular behavior. $\tau_{e}$ is the wall shear stress caused by blood pressure, which is expressed by $\tau_{e}=100-86\exp\left[-5000(\log_{10}{\left(\log_{10}P_{B}\right)}^{5.4}\right]$. $Q_{\mathrm{ref}}$ is the largest value of $Q_{B}$ in the microvascular network. $k_{m}$ and $k_{p}$ are positive constants with values of 0.07 [1/s] and 0.1 [1/s], respectively [16]. Shrinking tendency ($k_{s}$) is another parameter that represents the basal tendency of vessels to shrink in the absence of positive growth stimuli. $k_{s}$ is considered to be 0.35 [1/s] in this study [16]. Soltani and Chen [35] and Sefidgar et al. [16] described intravascular blood flow modeling in a dynamic network with more details.

#### 2.2.3. Interstitial Fluid Flow

Darcy’s law is a simplified form of the momentum equation, which can be applied for defining the fluid flow behavior in biological tissues as porous media like the tumor and normal tissues [23,52,53]:

(14) $${\vec{V}}_{i}=-k\nabla P_{i}$$

where ${\vec{V}}_{i}$, k, and $P_{i}$ show the interstitial fluid velocity (IFV), interstitium hydraulic conductivity, and IFP, respectively.

The incompressible steady state form of continuity equation with source and sink terms of biological tissues is expressed as follows [23,52]:

(15) $$\nabla .{\vec{V}}_{i}=\phi_{B}-\phi_{L}$$

$\phi_{B}$ shows the fluid flow rate per unit volume from or into the blood vessels. $\phi_{B}$ in the computational domain is calculated using Starling’s law anywhere there is a capillary network. Otherwise, $\phi_{B}$ is set at zero. $\phi_{L}$ demonstrates the fluid flow rate per unit volume from the interstitial space into the lymph vessels. It is worth mentioning that in this study, a uniform lymphatic system is considered only in the normal tissue [16]. Starling’s law is used to evaluate $\phi_{B}$. The general form of Equation (15) is as follows [16]:

(16) $$\nabla .{\vec{V}}_{i}=\frac{L_{p}S}{V}[{\bar{P}}_{B}-{\bar{P}}_{i}-\sigma_{s}(\pi_{B}-\pi_{i})]-\frac{L_{pL}S_{L}}{V}({\bar{P}}_{i})$$

$\frac{S}{V}$ shows the surface area of the vessel wall per unit volume of tissue. $L_{P}$, $\sigma_{s}$, $\pi_{B}$, and $\pi_{i}$ are the microvascular wall’s hydraulic conductivity, average osmotic reflection coefficient for plasma proteins, plasma osmotic pressure, and interstitial fluid osmotic pressure, respectively. $\frac{L_{pL}S_{L}}{V}$ is the product of hydraulic conductivity of the lymphatic vessel wall and surface area of the lymphatic wall per unit volume of tissue.

Combining Equations (14) and (16) with a constant value for k results in:

(17) $$-k\nabla^{2}P_{i}=\frac{L_{p}S}{V}[{\bar{P}}_{B}-{\bar{P}}_{i}-\sigma_{s}(\pi_{B}-\pi_{i})]-\frac{L_{pL}S_{L}}{V}({\bar{P}}_{i})$$

#### 2.2.4. Solute Transport 

Two transport mechanisms of convection and diffusion are considered in this study for describing drug delivery into the solid tumor as a porous medium [54]. Fick’s second law is used to show mass conservation as follow [23]:

(18) $$\frac{\partial C_{i}}{\partial t}+\nabla \cdot \vec{J}=0$$

where $C_{i}$ and $\vec{J}$ are solute concentration and solute mass flux, respectively.

Fick’s first law controls solute transport induced via the diffusion mechanism. Moreover, solute transport induced via the convection mechanism is obtained by multiplying the IFV by the solute concentration [16].

(19) $$\vec{J}=-D_{eff}\nabla C_{i}+{\vec{V}}_{i}C_{i}$$

where $D_{eff}$ shows the coefficient of the diffusion mechanism. By considering the source and sink terms of biological tissues and assuming a constant value for $D_{eff}$, the following equation shows the governing equation of solute transport [16,55].

(20) $$\frac{\partial C_{i}}{\partial t}=D_{eff}\nabla^{2}C_{i}-\nabla \cdot ({\vec{V}}_{i}C_{i})+\varphi_{B}-\varphi_{L}$$

φ_B_ shows the solute transport rate per unit volume from the blood vessels into the interstitial tissue. The following equation indicates $\varphi_{B}$ based on the model proposed by Patlak [56]:

(21) $$\varphi_{B}=\phi_{B}(1-\sigma_{f})C_{p}+\frac{PS}{V}(C_{P}-C_{i})\frac{Pe}{e^{Pe}-1}$$

where $\sigma_{f}$, $C_{P}$, and P show the filtration reflection coefficient, plasma solute concentration, and the coefficient of micro-vessel permeability, respectively. Pe shows the Peclet number with an equation of $\frac{\phi_{B}(1-\sigma_{f})V}{PS}$. 

$\varphi_{L}$ is the solute transport rate per unit volume from the interstitium to the lymphatic vessels, which are considered just in the normal tissue in the present study [16], with the following equation:

(22) $$\varphi_{L}=\phi_{L}C_{i}$$

### 2.3. Numerical Simulation Explanation

To clarify the numerical model applied in the present study, boundary and initial conditions, numerical modeling process, grid-independent solution, and parameter values are described in the following sections.

#### 2.3.1. Boundary and Initial Conditions 

In intravascular blood flow analysis, the blood pressure in the inlet and outlet of parent vessels is considered to be 25 mmHg and 10 mmHg [16].

In the interstitial fluid flow analysis, the IFP in the outer boundaries of normal tissue is considered to have a value of 0 Pa, which is the surrounding pressure in the present study [23]. For the boundary between tumor and normal tissues, the IFP and IFV are continuous, as follows:

(23) $$\begin{array}{l} P_{i}{|}_{R^{-}}=P_{i}{|}_{R^{+}}\\ -k_{t}{\nabla P_{i}|}_{R^{-}}=-k_{n}{\nabla P_{i}|}_{R^{+}} \end{array}$$

where $R^{-}$ and $R^{+}$ represent the radius of the tumor edge at the tumor and normal tissues, respectively. $k_{t}$ and $k_{n}$ show the hydraulic conductivity of the tumor and normal tissues, respectively [23]. 

In the solute transport analysis, an open boundary condition is applied in the normal tissue boundaries with an equation of $-n.\nabla C_{i}=0$. n depicts the normal vector. The continuity of solute concentration and its flux are considered in the inner boundary between the tumor and normal tissues [23]:

(24) $$\begin{array}{l} C_{i}{|}_{R^{-}}=C_{i}{|}_{R^{+}}\\ (D_{eff}{}^{t}\nabla C_{i}+{\vec{V}}_{i}C_{i}){|}_{R^{-}}=(D_{eff}{}^{n}\nabla C_{i}+{\vec{V}}_{i}C_{i}){|}_{R^{+}} \end{array}$$

The initial values of 0 mmHg and 10 mmHg are considered for IFP and blood intravascular pressure, respectively. An initial value of 12 μm is considered for the capillary vessel diameter. The initial value for solute concentration is considered equal to $0\;\;mol/m^{3}$. The bolus injection is modeled with the equation $C_{P}(t)=C_{0}e^{-t/\tau }$. $C_{0}$ shows the maximum amount of concentration in the plasma, which is $1\;\;mol/m^{3}$ in this study. τ shows the time constant.

#### 2.3.2. Numerical Modeling Process

**Figure 5:**
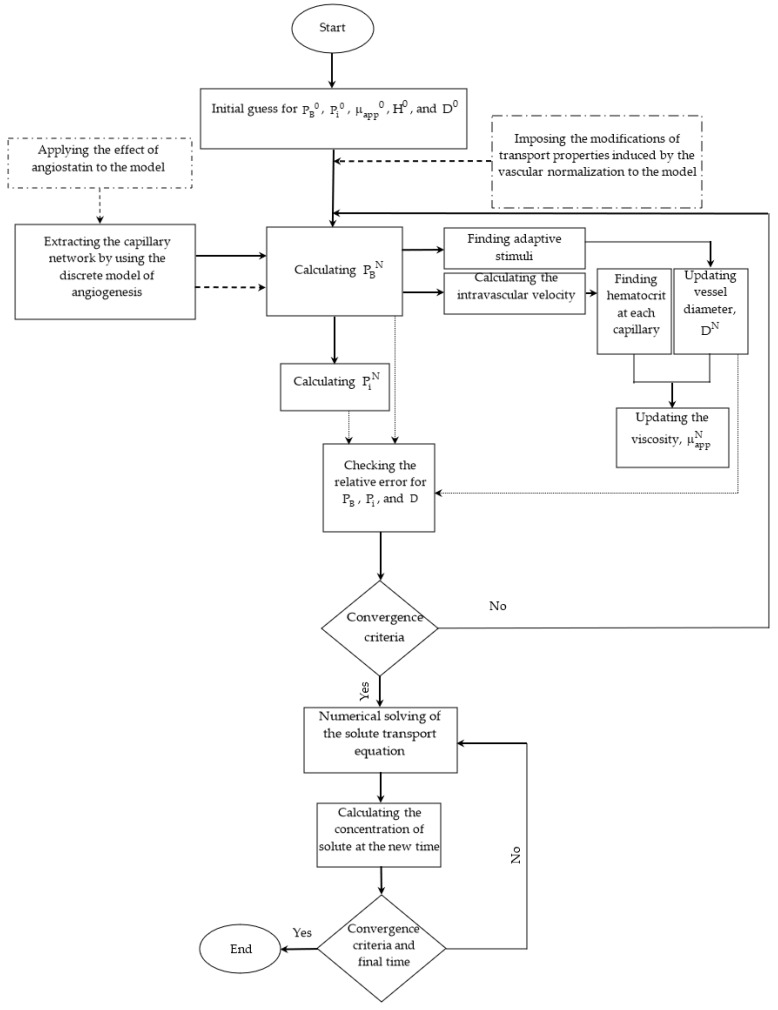
The flowchart of the numerical modeling procedure.

In this study, after considering the initial guesses, intravascular blood pressure (IBP) in each node is achieved by numerically solving the discretized form of Equation (5) via an iterative computational method. The obtained value for the intravascular pressure is used in the discretized form of Equation (17) for calculating the IFP. Intravascular flow and subsequently the stimuli of vessel diameter adaptation are found by determining the amount of intravascular pressure, so the structure of the capillary network is reconstructed. Hematocrit and blood viscosity are also updated. The updated values of capillary diameter and blood viscosity are used to calculate the IBP and subsequently the IFP. This iterative process continues until convergence is achieved. The relative error for this set of equations is defined as $\frac{X_{N}-X_{O}}{X_{O}}$, in which X shows $P_{B}$, $P_{i}$, and D. N and O depict the current step and previous step, respectively. The numerical solution of the equations continues until the relative error becomes less than $10^{-6}$. The obtained value for IBP is used to calculate the solute concentration in connection to the IFP (Equation (21)) by using the finite element method with a precision of $10^{-6}$ for the residual convergence criterion. Figure 5 shows the flowchart of the simulation procedure.

#### 2.3.3. Grid Independent Solution

The independence of results from the grid size is checked in different tumor sizes for both fluid flow and solute transport analyses. IFP, IFV, and C_i_ are evaluated by generating coarse grids and by making denser grids. This process continues until the difference between the two last results becomes negligible. 

#### 2.3.4. Parameters Value

Different values are considered for parameters of the interstitial fluid flow and solute transport analyses in different tissue types based on previous research [16,18,23,55,57,58,59]. The descriptions of all parameters are presented in Table 1. $L_{P}$ in tumor and normal tissues is considered based on previous calculations [60] and research [16,23,61]. k  is considered in the present study based on our previous works [16,23,61] for normal and tumor tissues. $\frac{S}{V}$ has a value of $70\;\frac{1}{cm}$ and $200\;\frac{1}{cm}$ in normal and tumor tissues, respectively [23]. $\sigma_{s}$ was obtained for normal tissue to be equal to 0.91 [62]. $\sigma_{s}$ is calculated using the spherical solute–cylindrical pore model for tumor and normalized tissues based on the previous studies [21,23]. $\pi_{B}$ and $\pi_{i}$ are considered to have the same values as our previous study [23]. In the present study, $P_{L}$ and $\frac{L_{PL}S_{L}}{V}$ are assumed just in the normal tissue, and their values are considered based on the work of Pishko et al. [57]. $D_{eff}$ and P for tumor and normal tissues are considered based on [23] for $F{\left(ab^{\prime }\right)}_{2}$. $\sigma_{f}$ for different tissues is calculated by using $\sigma_{f}={\left[1-{\left(1-\frac{Solute\;radius}{Pore\;radius}\right)}^{2}\right]}^{2}$ [63,64]. It is worth mentioning that vascular pore radius was considered to be 0.75 μm and 4.45 nm in tumor and normal tissues, respectively. A reduction of one-fifth in pore radius was applied due to the normalization induced by the anti-angiogenic therapy [23]. $\frac{S}{V}$ decreases by 42% after the anti-angiogenic therapy. A 68% reduction in vascular permeability ($P\frac{S}{V}$) occurs after normalization. $D_{eff}$ and k  are considered to be the same as before treatment. A 5-fold decrease in $L_{P}$ was measured after the anti-angiogenic treatment. More information can be found at [23].

## 3. Validation

**Figure 6:**
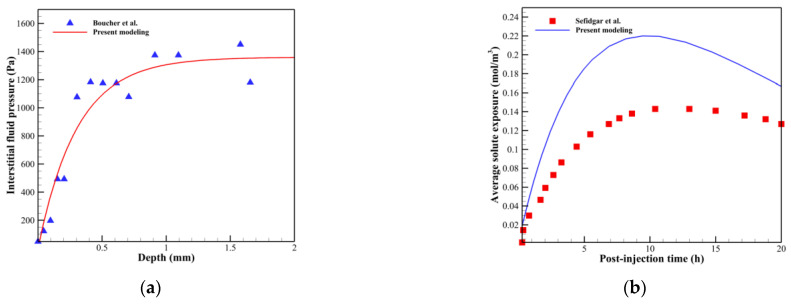
Comparison between results of the present model and literature [16,66]. (**a**) A comparison between the results of the interstitial fluid pressure distribution in the present study and the work of Boucher et al. [66]. (**b**) A comparison between the results of the average solute exposure in the present study and the work of Sefidgar et al. [16].

As the present study involves complex multi-scale and multi-physics modeling, it is not possible to validate it experimentally. Moreover, the present study addresses various complications, which contribute to its progressive nature. Therefore, to verify the present model, various phenomena from the case studies in the literature [16,28,66] are duplicated in this study. In the first stage, angiogenesis is modeled under the influence of endostatin, another anti-angiogenic agent, based on the study by Tee and DiStefano III [28], by administering a continuous injection with a dose of 20 mg/kg/day. As reported in our previous study [30], capillary growth toward the tumor was halted in this case study, which is consistent with the literature [28]. Experimental research has demonstrated a reduction in tumor microvascular density following anti-angiogenic therapy. For instance, Soto-Pantoja et al. [67] conducted an in vivo study, revealing a 50% decrease in vessel density in human A549 lung tumor xenografts subcutaneously implanted in mice following angiostatin injection. Similarly, a decrease in blood vessel density was observed in established ovarian cancer in mice after angiostatin treatment [68]. To verify the interstitial fluid flow behavior, the physical conditions of the experimental work by Boucher et al. [66] are imposed on the present study’s model, and the results show good agreement between the two research studies, as shown in Figure 6a. In the third stage, the average drug exposure is determined in R=0.4 cm, considering the same methodology as described in the previous study [16], while taking into account the microvasculature of the present research. The comparison is shown in Figure 6b. Upon comparing the results, it is evident that the solute accumulation process over time aligns well with the literature [16]. However, a discrepancy arises due to the variation in computational domains. Sefidgar et al. [16] considered a single parent vessel sprouting toward half of a circular tumor, leading to a different capillary network structure.

## 4. Results and Discussion

Drug delivery into solid tumors with different sizes and remodeled dynamic networks are investigated numerically in this study. The effect of the anti-angiogenic adjuvant treatment on the quality of drug delivery is studied here. Three cases are considered to study this effect. In case one, modification in response to the inhibitory effect of angiostatin and consequently updated tumor-induced microvascular network is considered without any change in the interstitial transport properties. In the second case, only modifications in transport properties are considered. In the third case, both modifications in the microvascular network and transport properties in response to the anti-angiogenic therapy are considered. 

Interstitial fluid flow is carried out to find out the IFP and IFV distributions. Solute transport analysis is considered to evaluate the concentration of the therapeutic agent delivered into the tumor site. Two parameters of non-dimensional average solute exposure (NDASE) and non-dimensional average solute distribution non-uniformity (NDASDNU) are introduced based on our previous research [23] as indicators of the quality of drug delivery into the tumor. 

As different tumor sizes are considered, the final time for each geometry is defined such that the average amount of solute in the tumor site reaches one percent of its maximum value after considering vascular normalization modification (case 3). This time is equal to 820,210 s, 709,150 s, 511,750 s, and 646,160 s in tumors with R=0.8 cm, R=0.6 cm, R=0.4 cm, and R=0.2 cm, respectively.

### 4.1. Fluid Flow Analysis

**Figure 7:**
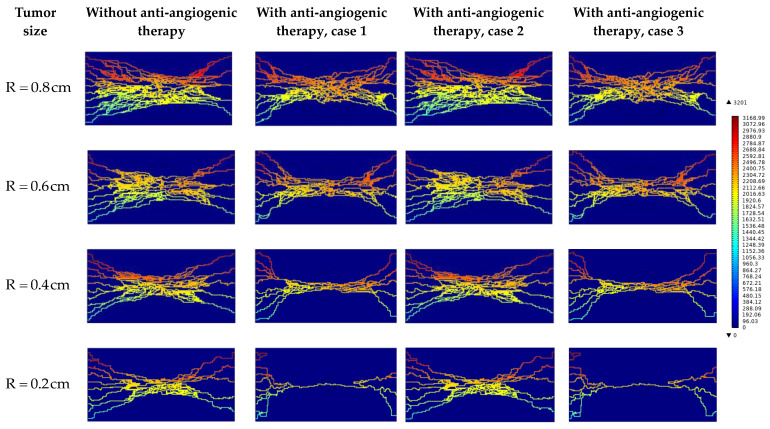
Non-dimensional IBP contour in different tumor sizes and states.

**Figure 8:**
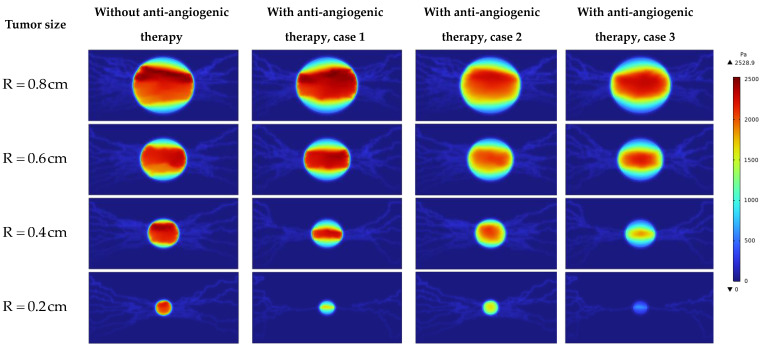
Non-dimensional IFP contour in different tumor sizes and states.

**Figure 9:**
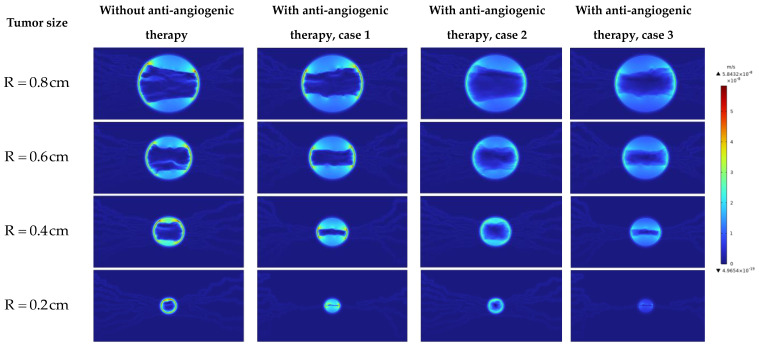
Non-dimensional IFV contour in different tumor sizes and states.

Investigating the interstitial fluid flow is significant because high IFP in the tumor site, its sudden decrease, and consequently the sudden increase of IFV at the tumor margin were introduced as a main barrier for qualified drug delivery in the literature [18,19,20,21,69]. Therefore, interstitial fluid flow analysis in connection with intravascular blood flow is performed. Figure 7, Figure 8 and Figure 9 demonstrate the non-dimensional distribution of IBP, IFP, and IFV (relative to their maximum value) in different case studies of the present research.

Figure 7 shows the non-dimensional IBP contour in different tumor sizes and states. It can be seen that the distribution of IBP is dependent on the microvascular network morphology in the first order and on the transport properties next.

**Figure 10:**
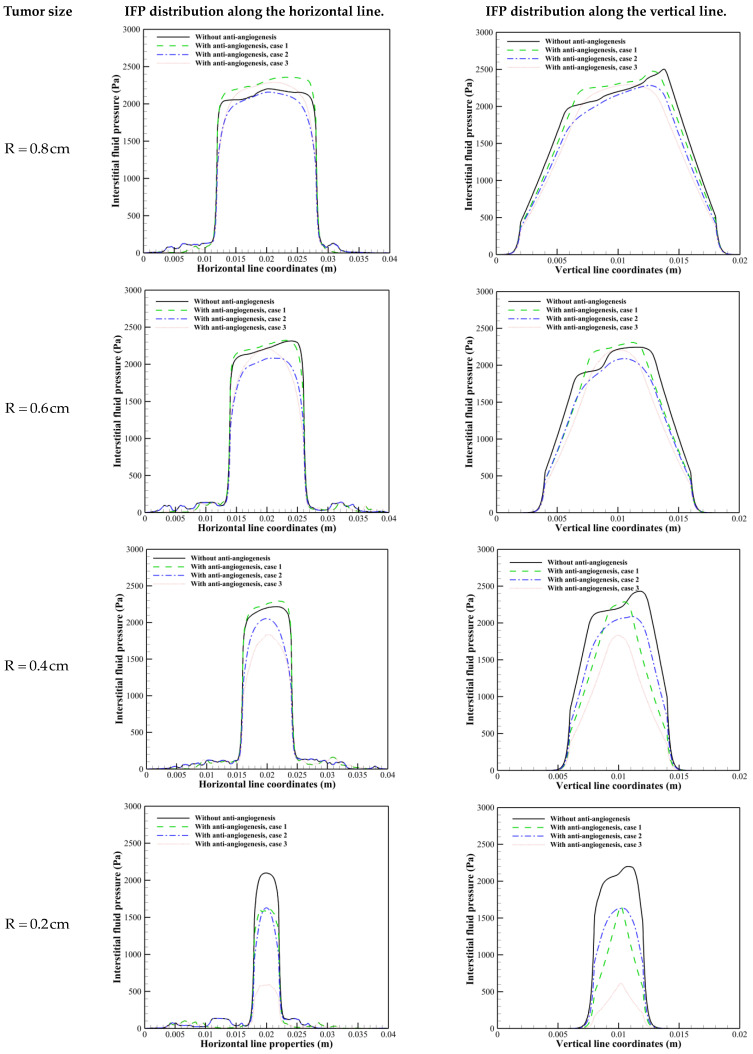
IFP distribution along horizontal and vertical cut lines in different tumor sizes.

Figure 8 and Figure 10 show the non-dimensional contour of IFP and IFP distribution along cut lines shown in Figure 1. According to these figures, the IFP in the tumor site is more than the surrounding normal tissue. There are three main reasons for this phenomenon: higher density of the microvascular network, more leakage of the capillaries, and lack of an efficient lymphatic system in the tumor site. It is obvious that IFP does not have a uniform distribution in the tumor site as what is in the macroscopic analysis. Because there is non-uniform distribution of the blood vessels and consequently non-uniform fluid flow sources ($\phi_{B}$ in Equation (15)). These results improve the visualization of the tumor microenvironment behavior and bring a more realistic view of that.

The value of the IFP in the tumor area before anti-angiogenic therapy in different tumor sizes studied in this research is in accordance with an experimental study by Butcher and Jain [70], who reported the tumor pressure range from 586 to 4200 Pa. In comparison to our previous study [23], the IFP has a higher value in the tumor site before considering the anti-angiogenic therapy. This result is in accordance with our previous research [16].

By comparing the IFP distribution before considering anti-angiogenesis and considering it by case 1 in Figure 8 and Figure 10, the significant effect of micro-vessels feeding the tumor is demonstrated. IFP distribution in R=0.8 cm and R=0.6 cm in Figure 8 and Figure 10 shows that even though the density of micro-vessels is reduced by 24% and 13%, respectively [30], in response to the inhibitory effect of angiostatin, the maximum amount of IFP occurs in approach 1 of considering anti-angiogenesis. This result shows that the interstitial fluid flow behavior is more dependent on the structure of the microvascular network than on its density. This behavior changes in R=0.2 cm by pruning severely micro-vessels, such that IFP has its maximum value in the case without considering the anti-angiogenic therapy.

It has been demonstrated preclinically [71] that anti-angiogenic therapy leads to the establishment of a pressure gradient across vasculature, as depicted in Figure 8 and Figure 10. It is evident that various approaches to modeling anti-angiogenesis result in the development of a pressure gradient between the microvasculature wall and its periphery. Another observation in the present study is the reduction of IFP induced by anti-angiogenic therapy, which has been reported in different experimental trials [71,72,73,74]. The IFP in tumors implanted subcutaneously in mice with an approximatively equivalent radius of 4 mm decreases ~50% after treatment with the anti-angiogenic agent EGCG [75]. The reduction in the average amount of IFP in the tumor with R = 0.4 cm is close to this value (~45%). However, this close agreement may be interpreted qualitatively rather than quantitatively, as different algorithms were utilized between the two studies.

**Figure 11:**
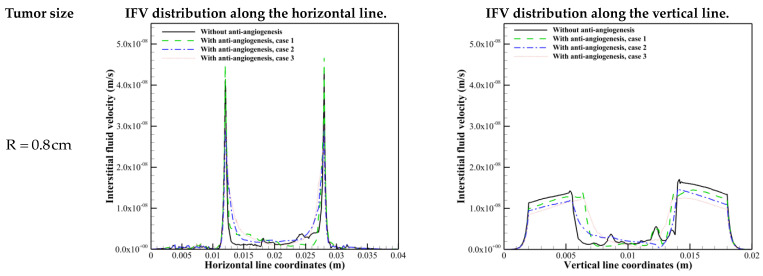

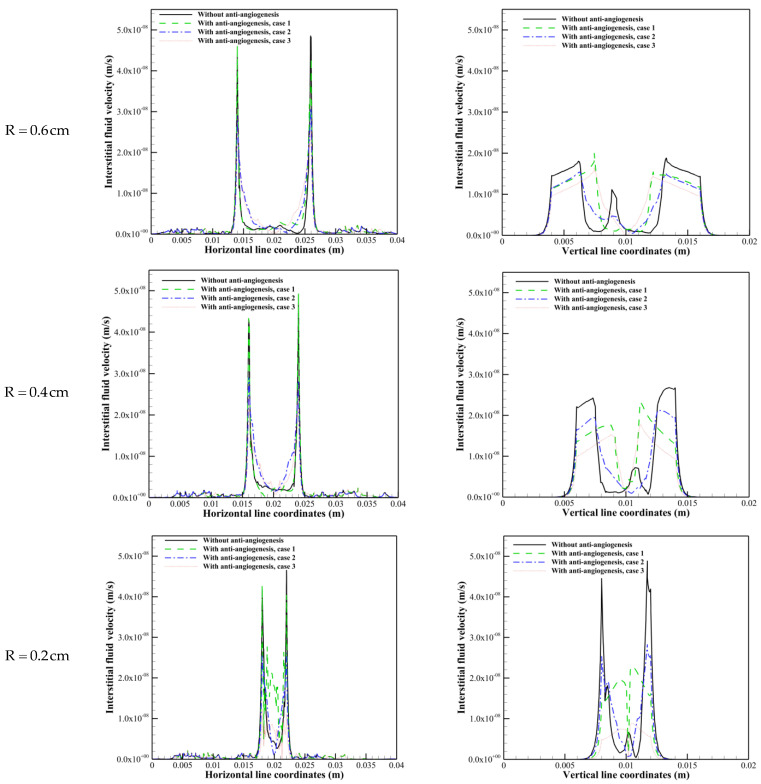
IFV distribution along horizontal and vertical cut lines in different tumor sizes.

Figure 9 and Figure 11 show the contour of IFV and its distribution along cut lines. In dissimilarity with macroscopic studies, IFV has a non-uniform distribution in tumor tissue. IFV has a non-zero value in some parts of tumor tissue, as IFV is proportional to the IFP gradient (Darcy’s law). Non-uniform IFP distribution, and consequently the existence of pressure gradient in the tumor site, results in non-zero IFV inside the tumor.

In Figure 11, in all tumor sizes, IFV along the horizontal line has its non-zero maximum value at the tumor margin before considering the anti-angiogenic treatment because of the almost uniform distribution of IFP along this line. However, IFV has a non-zero value not only in the tumor margin but also outside the dense region of micro-vessels along the vertical line.

In addition to the discussion made about the effect of anti-angiogenesis case 1 on interstitial fluid flow behavior, it is shown that other than R = 0.2 cm, in which anti-angiogenesis induced via angiostatin application causes a 55% decrease in microvascular network density [30], this approach (case 1) causes a pressure gradient in the inner areas only along the vertical direction. The modification in IFV distribution in R=0.2 cm is obvious. An intensive decrease in capillary density limits the source of flow, which is responsible for the decrease in IFP and non-zero IFV inside the tumor along both horizontal and vertical directions. Figure 10 shows that the second approach of anti-angiogenic treatment causes a decrease in IFP in all tumor sizes. Moreover, this approach modifies the steep pressure gradient in the tumor margin and shifts the pressure gradient to the inner areas. In accordance with the modification in IFP with the second approach, IFV behavior is also modified. The effect of normalization induced by the third approach on IFP in R=0.4 cm is shown in Figure 10 just by causing the IFP gradient, which causes non-zero IFV in inner areas and a decrease in IFV at the tumor margin. By decreasing the tumor size, the IFP decrease induced by the third approach of vascular normalization is increased.

### 4.2. Solute Transport Analysis

A single bolus injection, whose equation is described in Section 2.3.1, is considered in this study, and the convection-diffusion equation is solved numerically to find the concentration distribution of the therapeutic agent.

**Figure 12:**
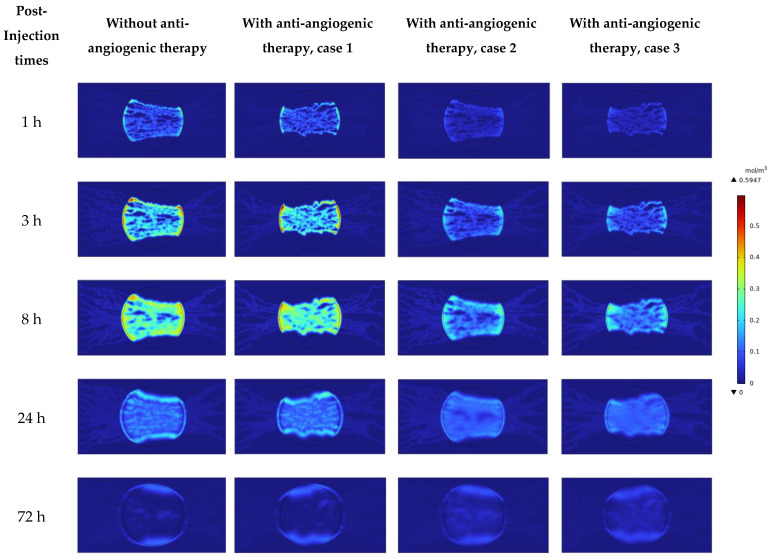
Non-dimensional solute concentration contour in R = 0.8 cm at different post-injection times.

Figure 12 shows the non-dimensional solute concentration contour in R=0.8 cm at different post-injection times. This figure is dimensionless relative to its maximum value. The non-uniform distribution of the solute is obvious, which is the result of the tortuous structure of microvasculature. As shown in Figure 12, vascular normalization modifies the drug wash-out phenomenon in the tumor periphery. This is evident from the modification of the spatial distribution range toward the inner parts of the tumor (case 1), and moreover a reduction in the amount of drug wash-out (cases 2 and 3) compared to the pre-anti-angiogenic therapy. 

By considering the modifications in transport properties (cases 2 and 3), while the maximum amount of solute reaching the tumor site is decreased, the tumor is exposed to the drug for a longer period of time. This behavior causes more uniformity of solute distribution because the mechanism of diffusion in the interstitium has more time for carrying solute to the sites with less or no density of micro-vessels. It is observed in Figure 12 that the uniformity in the solute distribution in the entire tumor region at 72 h post-injection is higher in cases 2 and 3 compared to the case without considering anti-angiogenic therapy.

**Figure 13:**
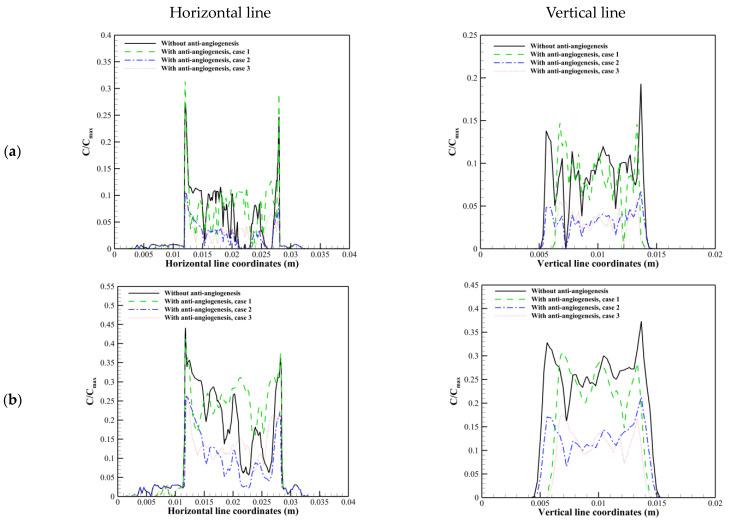

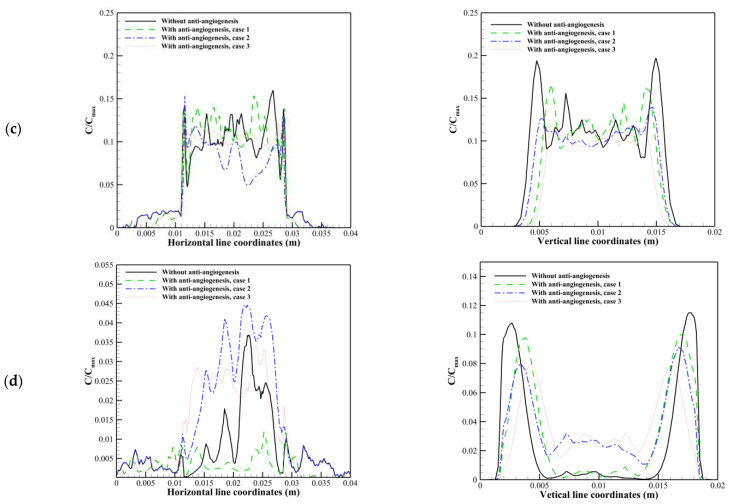
Solute distribution along horizontal and vertical cut lines in R=0.8 cm. (**a**) 1 h post-injection. (**b**) 8 h post-injection. (**c**) 24 h post-injection. (**d**) 72 h post-injection.

Figure 13 shows the distribution of solute along horizontal and vertical cut lines in R=0.8 cm at different post-injection times. It is obvious that the solute has a heterogeneous distribution in the tumor region because of the heterogeneity in micro-vessels distribution as the channels for transporting the therapeutic agents into the tumor. By comparing the present study’s results with previous ones, which assumed a uniform distribution of blood vessels in vital regions of tumors, it is obvious that considering a dynamic microvascular network based on real phenomena is essential to have a more realistic view.

The solute concentration has its jumped wash-outed maximum value in the tumor margin along the horizontal line before considering anti-angiogenesis treatment because of a sudden increase in IFV profile in the margin (Figure 13a). The first approach of anti-angiogenesis treatment cannot modify this behavior because it cannot modify the IFP and IFV distribution in R=0.8 cm along the horizontal line in Figure 10 and Figure 11. The solute concentration along the vertical line has its maximum value in the inner areas of the tumor at early injection (1 h). Over time, the solute concentration starts to wash out from the tumor boundaries because of the out-flow convective mechanism. Vascular normalization induced via anti-angiogenesis modifies this behavior at each post-injection time (Figure 13b–d, vertical line).

The solute concentration increases fast before anti-angiogenic therapy. Then, the concentration starts to decrease (as seen in Figure 13c) because of plasma clearance. Vascular normalization, especially by the second and third approaches, makes a difference in the timing of drug delivery by modifying the transport properties (which is discussed in detail in our previous study [23]) or modifying both transport properties and micro-vessels distribution.

**Figure 14:**
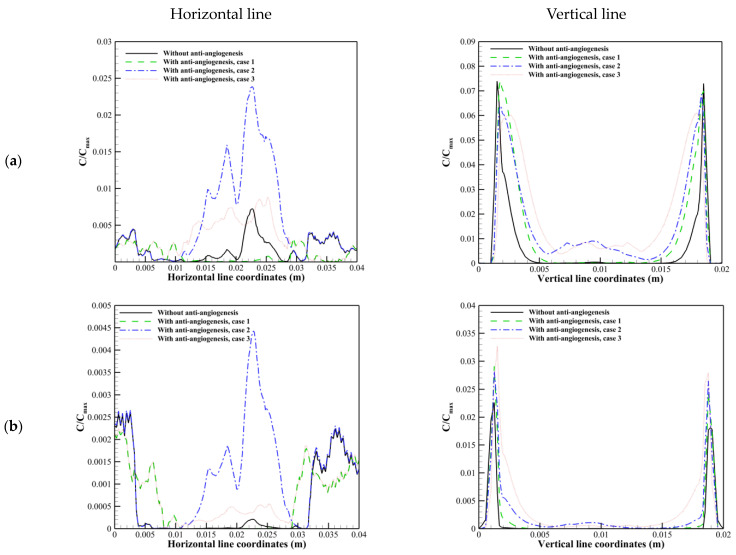
Solute distribution along horizontal and vertical cut lines in R=0.8 cm. (**a**) 120 h post-injection. (**b**) 216 h post-injection.

It is shown in Figure 13 that at 24 h post-injection, drug exposure and uniformity are improved by vascular normalization, especially by the second and third approaches. This phenomenon is more obvious at 72 h post-injection. This behavior is related to (a) the trade-off between P, $\frac{S}{V}$, and $C_{P}$, which control the trans-vascular diffusion as the dominant transport mechanism in areas with dens micro-vessels, and (b) modifications in trans-vascular convection and interstitium convection transport mechanisms (via the modifications in IFP, IFV, $L_{p}$, and $\frac{S}{V}$), which play critical roles in drug delivery in areas with lower micro-vessels density and tumor margins. In other words, by modifying transport properties (case 2) and also the microvascular network morphology (case 3) due to vascular normalization, the tumor exposure to the therapeutic agent occurs slowly, and subsequently, plasma clearance does not occur fast like the untreated tumor. Therefore, the tumor is exposed to the drug for a longer time (it is shown in Figure 14 that at 120 h and 216 h post-injection, in contrast to the untreated tumor and the first approach used for anti-angiogenesis, the second and third approaches of vascular normalization cause the presence of solute inside the tumor region long time after a single injection).

Based on the aforementioned discussion, the type of therapeutic agent used in either chemotherapy alone or in combination with anti-angiogenic treatment is another crucial factor that governs the effectiveness of drug delivery and vascular normalization. In further detail, the type of therapeutic agent through its molecular weight is a determinant of the plasma clearance (which its rate is expressed by drug half-life [76]), osmotic filtration reflection coefficient, effective diffusion coefficient, and micro-vessel permeability coefficient [18]. For example, a therapeutic agent with a rapid plasma clearance half-life results in a faster decrease in concentration within the tumor interstitium. Consequently, the interstitium has a shorter window of exposure to it. Vascular normalization induced via anti-angiogenic therapy reduces the excessive leakiness of the microvascular network [77,78] and may improve the quality of drug delivery. As outlined in our prior study [23], vascular normalization has the potential to improve clearance behavior by directly modifying the parameters governing trans-vascular diffusion, trans-vascular convection, and interstitial convection and indirectly influencing the interstitial diffusion transport mechanism. In other words, modification of transport properties and microvascular network induced via vascular normalization can cause an improvement in solute distribution via a regulated process of drug entry into the interstitium and subsequent return to the plasma. Thus, during a specific perfusion time frame, precise intensities of vascular normalization have the potential to enhance the delivery of a specific therapeutic agent to a solid tumor with predefined properties. More discussion can be found in [23]. Thus, it is important to study effective parameters in drug delivery, with the specific plasma clearance half-life of the therapeutic agent being one of them.

It is observed that the first approach considered for anti-angiogenesis can decrease drug wash-out (along the vertical axis) due to the modification in the distribution of blood micro-vessels caused by the inhibitory effect of angiostatin. However, this approach cannot improve the distribution of solute.

The values of two parameters, NDASE and NDASDNU, are determined for different tumor sizes and under various vascular normalization approaches at different final time durations. The results are reported in Table 2. In terms of both average drug exposure and uniformity of drug exposure, the first approach considered to mimic anti-angiogenesis does not yield a positive effect. Since the blood micro-vessels transfer drugs to the tumor site, updating the micro-vessel distribution due to the inhibitory effect of angiostatin not only leads to a reduction in microvascular density but also concentrates them in central areas of the tumor. This, in turn, increases non-uniformity in drug distribution without significantly altering exposure. The non-uniformity increases as the tumor size decreases because of the increase in micro-vessel pruning due to the inhibitory effect of angiostatin. This demonstrates that the heterogeneous distribution of micro-vessels results in a non-uniform distribution of therapeutic agents at the tumor site, transported via trans-vascular diffusion and convection mechanisms via blood vessel sources. On the contrary, it has been demonstrated that a more uniform distribution of the microvascular network leads to less non-uniformity in solute distribution, as demonstrated in R=0.2 cm, even before considering anti-angiogenesis in the present study. Clinical studies demonstrate that tumor vessels exhibit significant heterogeneity in their distribution, diameter, density, and serpentine shape. This leads to low and heterogeneous blood flow in tumor tissue. The primary factors responsible are the mechanical forces generated via fluid and solid stress, along with an excess of vessel permeability [79]. Thus, reengineering the abnormal and heterogeneous microenvironment of solid tumors via approaches such as normalizing tumor blood vessels and the extracellular matrix, and alleviating vessel compression is conducted to overcome the challenges associated with cancer heterogeneity [39,77,78]. From another point of view, this behavior shows the determinative effect of the architecture of the microvascular network, which can be affected by the anti-angiogenic agent. So, the type of anti-angiogenic agent, as a controlling factor of pruning the micro-vessels and consequently the uniformity of their distribution, is an important factor in reengineering the microvascular distribution.

In addition to the microvasculature update, considering the modifications in transport properties (case 3) modifies the non-uniformity caused by case 1. This is due to the earlier discussion regarding prolonged tumor exposure to the therapeutic agent and improvements in IFP and IFV induced by the second approach of anti-angiogenesis. In tumor size R=0.8 cm, where microvascular network density is decreased by 13% [30], and, of even greater importance, micro-vessel morphology is modified to a more uniform distribution, anti-angiogenesis via the third approach increases drug uniformity by 7%. 

The results indicate that the second approach of anti-angiogenesis improves drug distribution by 19%, 17.3%, 14.1%, and 38.7% in R=0.8 cm, R=0.6 cm, R=0.4 cm, and R=0.2 cm, respectively, without causing a significant difference in average drug exposure compared to before anti-angiogenesis. In this case, the microvascular network suppression is not considered, instead transvascular diffusion and convection mechanisms are improved in areas with a blood source due to the modification of IFP and IFV, as demonstrated in Figure 10 and Figure 11. Furthermore, the modification of IFV due to the establishment of an IFP gradient in the inner areas of the tumor, achieved via the second approach of anti-angiogenesis, improves the interstitial convection mechanism. All of these factors contribute to a greater uniformity in the distribution of the therapeutic agent. The average drug exposure does not change significantly because, although applying anti-angiogenesis initially decreases drug exposure, it increases afterward. The experimental evidence supports the notion that when used as an adjuvant treatment alongside basic therapies such as chemotherapy and radiotherapy, antiangiogenic treatment can lead to a more uniform distribution of therapeutic agents within the tumor [80,81].

The greatest increase in uniformity occurs in R=0.2 cm. This is primarily because the entire tumor site is supplied by blood micro-vessel sources, resulting in uniform delivery via transvascular mechanisms. Furthermore, modifying the interstitial fluid flow improves the role of interstitial convection in this size. This result highlights the strong dependency of drug delivery on the microvascular network structure, or to be more precise, on the distribution and density of micro-vessels.

The modification of solute distribution in different time windows varies depending on the specific cases considered for anti-angiogenesis simulation in this study. However, based on the results of NDASE and NDASDNU across different tumor sizes, the second approach to anti-angiogenic therapy can be interpreted as the most effective method for enhancing the quality of drug delivery in this current research. This illustrates the significant impact of modifying transport properties via vascular normalization on the response to the therapeutic agent. However, the decision regarding the effectiveness of the adjuvant anti-angiogenic treatment strategy cannot be accurate without considering the importance of the uniformity of the microvascular structure at the tumor site. This is highly dependent on the type of anti-angiogenic agent. Therefore, in order to gain a comprehensive understanding of the effectiveness of anti-angiogenic therapy, factors beyond those previously discussed, such as tumor size, concurrent therapeutic agent type, and time course of perfusion [23], the type of anti-angiogenic agent and its role in normalizing the microenvironment are crucial.

Based on the results, it should be emphasized that different parameters of anti-angiogenic agent type, concurrent therapeutic agent type, and tumor geometric and physical characteristics are determinative factors that specify the efficiency of anti-angiogenic adjuvant therapy. Therefore, quantitative results obtained from the present study, such as the percentage of improvement in drug delivery induced via anti-angiogenic therapy and the proposal of the most effective approach to vascular normalization, are dependent on the aforementioned factors. However, Qualitative findings from the present study emphasize the crucial influence of microvascular network structure. This underscores the importance of selecting the most appropriate anti-angiogenic strategy, one that optimizes the distribution and density of the microvascular network and response of interstitial fluid flow and solute transport properties to anti-angiogenic therapy, rather than merely suppressing micro-vessels. These insights can potentially be applied to other types of tumors, where drug delivery can be simulated using the numerical model employed in this study. In other words, the findings of the current research can open a new horizon on the dual function of the tumor microvascular network and how to take advantage of anti-angiogenic adjuvant treatment in improving the quality of drug delivery into the tumor.

It is important to mention that this study was not experimentally validated due to constraints related to available laboratory resources. To rely more quantitatively on the results of the present study, the numerical model could be integrated into experimental studies. There exists experimental research that assesses the combination therapy of chemotherapy and anti-angiogenic therapy [8,9,10,82,83,84]. However, developing an experimental study to investigate the details of tumor microvasculature considered in the current numerical model could be achieved by examining the effect of anti-angiogenic therapy on drug delivery to tumor fragments or cells implanted in the rat cornea [85,86,87]. Furthermore, the impact of anti-angiogenic agents on the quality of chemotherapy could be assessed in vivo using a zebrafish model [88,89]. Another potential platform for examining the tumor, its microvasculature, and methodologies for its therapy is via development in vitro using microfluidic systems [90,91].

## 5. Conclusions

In this study, a multi-scale numerical model, ranging from cell to tissue level, is developed to explore the impact of vascular normalization on drug delivery within a dynamic solid tumor microvasculature. This paper integrates mathematical models of intravascular blood flow and interstitial fluid flow to compute IFP, IFV, and IBP. These values are then applied to the convection-diffusion equation to simulate the solute distribution in different tumor sizes while considering various vascular normalization approaches. 

It is shown that the interstitial fluid and solute are distributed heterogeneously, a result of the heterogeneous distribution of micro-vessels. The results demonstrate a high dependency of IFP, IFV, and solute distributions on the microvasculature structure (distribution and density). Consequently, the impact of vascular normalization on drug delivery is greatly influenced by the microvascular structure.

The results demonstrate the effectiveness of all vascular normalization approaches in correcting drug wash-out from the tumor margins. This is achieved by modifying convection as the dominant transport mechanism of drug delivery in the tumor margin.

The results of the first approach to vascular normalization show that the inhibitory effect of angiostatin in suppressing the microvasculature and reducing its density cannot lead to an improvement in drug delivery to the tumor site in terms of both average drug exposure and its uniformity. In other words, this paper illustrates the function of the tumor capillary network as a double-edged sword. On one hand, it disrupts drug delivery; on the other hand, it aids in delivering the drug to the tumor site. This is demonstrated using a model that incorporates more realistic considerations in the present study for the first time. Modifying the transport properties accompanied by the microvasculature reform caused by vascular normalization in the third approach results in improving the outcomes of the first approach by improving the drug delivery schedule via modifications in solute transport mechanisms.

According to the results, it can be concluded that the effect of the anti-angiogenic agent is vital in influencing drug delivery in combination therapy. This is because the distribution and density of the microvascular network are highly dependent on the mechanism of action of the anti-angiogenic agent. Therefore, choosing the most appropriate strategy for deriving benefits from anti-angiogenic therapy is one of the most important factors in combination therapy with chemotherapy and anti-angiogenic therapy.

The second approach to anti-angiogenic therapy results in an improvement in drug distribution uniformity. This improvement is contingent on the microvascular distribution and density within a specific tumor size, indicated as the uniformity of capillary distribution. In other words, the benefit of drug delivery via anti-angiogenic adjuvant therapy, resulting from modifications in transport properties that extend the tumor’s exposure to the drug, will be most pronounced when the tumor is supplied by a more uniformly distributed capillary network. This scenario arises in R=0.2 cm, as observed in the second approach of anti-angiogenic therapy, considering the physics and conditions outlined in this paper, resulting in a 39% increase in drug exposure uniformity.

## Figures and Tables

**Table 1:** Interstitial fluid flow and solute transport properties for tumor, normalized, and normal tissues.

Parameter	Description	Normal Tissue	Normalized Tissue	Tumor Tissue	Reference(s)
$L_{p}\;(\frac{cm}{s\;mmHg})$	Hydraulic conductivity of the microvascular wall	$3.6\times 10^{-8}$	$5.6\times 10^{-8}$	$2.8\times 10^{-7}$	[23,55,61,65]
$k\;(\frac{cm^{2}}{s\;mmHg})$	Hydraulic conductivity of the interstitium	$8.53\times 10^{-9}$	$4.13\times 10^{-8}$	$4.13\times 10^{-8}$	[16,23,55,61]
$\frac{S}{V}\;(\frac{cm^{2}}{cm^{3}})$	Surface area of vessel wall per unit volume of tissue	70	116	200	[23]
$\pi_{B}(mmHg)$	Osmotic pressure of the plasma	20	19.2	19.8	[23]
$\pi_{i}(mmHg)$	Osmotic pressure of the interstitial fluid	10	15.1	17.3	[23]
$\sigma_{s}$	Average osmotic reflection coefficient for plasma proteins	0.91	$2.1\times 10^{-3}$	$8.7\times 10^{-5}$	[23]
$P_{L}(mmHg)$	Hydrostatic pressure of the lymphatics	0	-	-	[57]
$\frac{L_{PL}S_{L}}{V}(\frac{1}{s\;mmHg})$	Product of hydraulic conductivity of the lymphatic wall and surface area of lymphatic wall per unit volume of tissue	$1.33\times 10^{-5}$	-	-	[57]
$\sigma_{f}$	Osmotic filtration reflection coefficient	0.9	$2.06\times 10^{-3}$	$8.41\times 10^{-5}$	[23]
$D_{eff}(\frac{cm^{2}}{s})$	Effective diffusion coefficient	$0.16\times 10^{-8}$	$2\times 10^{-8}$	$2\times 10^{-8}$	[58]
$P(\frac{cm}{s})$	Micro-vessel permeability coefficient	$2.2\times 10^{-8}$	$9.54\times 10^{-8}$	$17.3\times 10^{-8}$	[23]
τ(hr)	Drug time constant	6.06	6.06	6.06	[18]

**Table 2:** Non-dimensional average solute exposure (NDASE) and non-dimensional average solute distribution non-uniformity (NDASDNU) in different tumor sizes and vascular normalization approaches. ↑ demonstrates the increase, and ↓ demonstrates the decrease.

Tumor Size	Without Anti-Angiogenic Therapy	With Anti-Angiogenic Therapy, Case 1	With Anti-Angiogenic Therapy, Case 2	With Anti-Angiogenic Therapy, Case 3
R=0.8 cm	NDASE: 0.02636	NDASE: 0.02697 (∼2.3%↑)	NDASE: 0.02653 (∼0.6485%↑)	NDASE: 0.02683 (∼1.78%↑)
	NDASDNU: 0.020417	NDASDNU: 0.024195 (∼18.5%↑)	NDASDNU: 0.016544 (∼19%↓)	NDASDNU: 0.018968 (∼7.1%↓)
R=0.6 cm	NDASE: 0.030992	NDASE: 0.031093 (∼0.32%↑)	NDASE: 0.030656 (∼1.1%↓)	NDASE: 0.030695 (∼0.96%↓)
	NDASDNU: 0.018542	NDASDNU: 0.026972 (∼45.46%↑)	NDASDNU: 0.01533 (∼17.3%↓)	NDASDNU: 0.022049 (∼18.91%↑)
R=0.4 cm	NDASE: 0.043371	NDASE: 0.042542 (∼1.9%↓)	NDASE: 0.042871 (∼1.15%↓)	NDASE: 0.041997 (∼3.16%↓)
	NDASDNU: 0.015275	NDASDNU: 0.036058 (∼136%↑)	NDASDNU: 0.01312 (∼14.1%↓)	NDASDNU: 0.032458 (∼112.5%↑)
R=0.2 cm	NDASE: 0.034	NDASE: 0.0351 (~3%↑)	NDASE: 0.03339 (∼1.9%↓)	NDASE: 0.034520 (∼1.4%↑)
	NDASDNU: 0.007138	NDASDNU: 0.025436 (∼256%↑)	NDASDNU: 0.004374 (∼38.7%↓)	NDASDNU: 0.023 (∼223%↑)

## Data Availability

The data presented in this research are available on request from the corresponding author.

## References

[1] Bodzioch M., Bajger P., Foryś U. (2021). Angiogenesis and Chemotherapy Resistance: Optimizing Chemotherapy Scheduling Using Mathematical Modeling. J. Cancer Res. Clin. Oncol. Vol..

[2] FKashkooli M., Souri M., Tavakkoli J.J., Kolios M.C. (2023). A Spatiotemporal Computational Model of Focused Ultrasound Heat-Induced Nano-Sized Drug Delivery System in Solid Tumors. Drug Deliv..

[3] Jain R.K. (1998). The Next Frontier of Molecular Medicine: Delivery of Therapeutics. Nat. Med..

[4] II D.A.H., Phillips C.M., Wu C., Lima E.A.B.F., Lorenzo G., Jha P.K., Jarrett A.M., Oden J.T., Yankeelov T.E. (2021). Biologically-Based Mathematical Modeling of Tumor Vasculature and Angiogenesis via Time-Resolved Imaging Data. Cancers.

[5] Rajora A.K., Ravishankar D., Zhang H., Rosenholm J.M. (2020). Recent Advances and Impact of Chemotherapeutic and Antiangiogenic Nanoformulations for Combination Cancer Therapy. Pharmaceutics.

[6] Ciccolini J., Benzekry S., Lacarelle B., Barlési F. (2015). Improving Efficacy of the Combination Between Antiangiogenic and Chemotherapy: Time for Mathematical Modeling Support(Letter). Proc. Natl. Acad. Sci. USA.

[7] Folkman J. (1971). Tumor Angiogenesis: Therapeutic Implications. N. Engl. J. Med..

[8] Yoshizawa Y., Ogawara K.-I., Fushimi A., Abe S., Ishikawa K., Araki T., Molema G., Kimura T., Higaki K. (2012). Deeper Penetration into Tumor Tissues and Enhanced in vivo Antitumor Activity of Liposomal Paclitaxel by Pretreatment with Angiogenesis Inhibitor SU5416. Mol. Pharmaceut..

[9] Gremonprez F., Descamps B., Izmer A., Vanhove C., Vanhaecke F., De Wever O., Ceelen W. (2015). Pretreatment with VEGF(R)-Inhibitors Reduces Interstitial fluid pressure, Increases Intraperitoneal Chemotherapy, Drug Penetration, and Impedes Tumor Growth in a Mouse Colorectal Carcinomatosis Model. Oncotarget.

[10] Escorcia F.E., Henke E., McDevitt M.R., Villa C.H., Smith-Jones P., Blasberg R.G., Benezra R., Scheinberg D.A. (2010). Selective Killing of Tumor Neovasculature Paradoxically Improves Chemotherapy Delivery to Tumors. Cancer Res..

[11] Majidpoor J. (2021). Mortezaee, Angiogenesis as a Hallmark of Solid Tumors-Clinical Perspectives. Cell. Oncol..

[12] Liang Q., Zhou L., Li Y., Liu J., Liu Y. (2022). Nano Drug Delivery System Reconstruct Tumour Vasculature for The Tumour Vascular Normalisation. J. Drug Target..

[13] Liang P., Ballou B., Lv X., Si W., Bruchez M.P., Huang W., Dong X. (2021). Monotherapy and Combination Therapy Using Anti-Angiogenic Nanoagents to Fight Cancer. Adv. Mater..

[14] Jafari-Matanagh S., Razavi S.E., Bonab M.B.E., Omidian H., Omidi Y. (2023). Multi-Dimensional Modeling of Nanoparticles Transportation from Capillary Bed into the Tumor Microenvironment. Comput. Biol. Med..

[15] Nikmaneshi M.R., Firoozabadi B., Mozafari A., Munn L.L. (2020). A Multi-Scale Model for Determining the Effects of Pathophysiology and Metabolic Disorders on Tumor Growth. Sci. Rep..

[16] Sefidgar M., Soltani M., Raahemifar K., Sadeghi M., Bazmara H., Bazargan M., Nayinian S.M.M. (2015). Numerical Modeling of Drug Delivery in a Dynamic Solid Tumor Microvasculature. Microvasc. Res..

[17] Hadjicharalambous M., Wijeratne P.A., Vavourakis V. (2021). From Tumour Perfusion to Drug Delivery and Clinical Translation of in Silico Cancer Models. Methods.

[18] Baxter L.T., Jain R.K. (1989). Transport of Fluid and Macromolecules in Tumors, I. Role of Interstitial Pressure and Convection. Microvasc. Res..

[19] Baxter L.T., Jain R.K. (1990). Transport of Fluid and Macromolecules in Tumors II. Role of Heterogeneous Perfusion and Lymphatics. Microvasc. Res..

[20] Baxter L.T., Jain R.K. (1991). Transport of Fluid and Macromolecules in Tumors III Role of Binding and Metabolism. Microvasc. Res..

[21] Jain R.K., Tong R.T., Munn L.L. (2007). Effect of Vascular Normalization by Antiangiogenic Therapy on Interstitial Hypertension, Peritumor Edema, and Lymphatic Metastasis: Insights from a Mathematical Model. Cancer Res..

[22] Mohammadi M., Aghanajafi C., Soltani M., Kilgour D.M., Kunze H., Makarov R., Melnik R., Wang X. (2021). Numerical Modelling of Drug Delivery in an Isolated Solid Tumor under the Influence of Vascular Normalization. Recent Developments in Mathematical, Statistical, and Computational Sciences in: AMMCS 2019, Springer Proceedings in Mathematics & Statistics.

[23] Mohammadi M., Aghanajafi C., Soltani M., Raahemifar K. (2022). Numerical Investigation on the Anti-Angiogenic Therapy-Induced Normalization in Solid Tumors. Pharmaceutics.

[24] Mohammadi M., Aghanajafi C., Soltani M. (2022). Simulation of the Role of the Anti-Angiogenic Therapy in Fluid Flow Behavior and Macromolecule Transport into a Heterogeneous Solid Tumor. Amirkabir J. Mech. Eng..

[25] Anderson A.R.A., Chaplain M.A.J. (1998). Continuous and Discrete Mathematical Models of Tumor-induced Angiogenesis. Bull. Math. Biol..

[26] Anderson A.R.A., Chaplain M.A.J., McDougall S.R., Jackson T.L. (2012). A Hybrid Discrete-Continuum Model of Tumour Induced Angiogenesis. Modeling Tumor Vasculature.

[27] Wu J., Long Q., Xu S., Padhani A.R. (2009). Study of Tumor Blood Perfusion and its Variation due to Vascular Normalization by Anti-Angiogenic Therapy based on 3D Angiogenic Microvasculature. J. Biomech..

[28] Tee D., DiStefano J. (2004). Simulation of Tumor-Induced Angiogenesis and its Response to Anti-Angiogenic Drug Treatment: Mode of Drug Delivery and Clearance Rate Dependencies. J. Cancer Res. Clin. Oncol..

[29] Zhao G., Chen E., Yu X., Cui H., LV J., Wu J. (2017). Three-Dimensional Model of Metastatic Tumor Angiogenesis in Response to Anti-Angiogenic Factor Angiostatin. J. Mech. Med. Biol..

[30] Mohammadi M., Soltani M., Aghanajafi C., Kohandel M. (2023). Investigation of the Evolution of Tumor-Induced Microvascular Network under the Inhibitory Effect of Anti-Angiogenic Factor, Angiostatin: A Mathematical Study. Math. Biosci. Eng..

[31] Mohammadi M., Sefidgar M., Kashkooli F.M., Aghanajafi C., Soltani M. Mathematical Modeling of the Effect of Angiostatin on the Density of the Circular Tumor-Induced Microvascular Network, In Proceedings of the 29th National and 7th International Iranian Conference on Biomedical Engineering (ICBME), Tehran, Iran, 22–23 December 2022.

[32] Stéphanou, Angélique, McDougall S.R., Anderson A.R.A., Chaplain M.A.J. (2006). Mathematical Modelling of the Influence of Blood Rheological Properties upon Adaptative Tumour-Induced Angiogenesis. Math. Comput. Model..

[33] McDougall S.R., Anderson A.R.A., Chaplain M.A.J. (2006). Mathematical Modelling of Dynamic Adaptive Tumour-Induced Angiogenesis: Clinical Implications and Therapeutic Targeting Strategies. J. Theor. Biol..

[34] Moath A., Xiao Y.X. (2021). The Influence of Tumour Vasculature on Fluid Flow in Solid Tumours: A Mathematical Modelling Study. Biophys. Rep..

[35] Soltani M., Chen P. (2013). Numerical Modeling of Interstitial Fluid Flow Coupled with Blood Flow through a Remodeled Solid Tumor Microvascular Network. PLoS ONE.

[36] Wu J., DING Z.-R., Cai Y., Xu S., Zhao G., Long Q. (2011). Simulation of Tumor Microvasculature and Microenvironment Response to Anti-Angiogenic Treatment by Angiostatin and Endostatin. Appl. Math. Mech..

[37] Zhao G., Yan W., Chen E., Yu X., Cai W. (2013). Numerical Simulation of the Inhibitory Effect of Angiostatin on Metastatic Tumor Angiogenesis and Microenvironment. Bull. Math. Biol..

[38] Ozturk D., Yonucu S., Yilmaz D., Unlu M.B. (2015). Influence of Vascular Normalization on Interstitial Flow and Delivery of Liposomes in Tumors. Phys. Med. Biol..

[39] Stylianopoulos T., Jain R.K. (2013). Combining Two Strategies to Improve Perfusion and Drug Delivery in Solid Tumors. Proc. Natl. Acad. Sci. USA.

[40] Steuperaert M., Debbaut C., Carlier C., De Wever O., Descamps B., Vanhove C., Ceelen W., Segers P. (2019). A 3D CFD Model of the Interstitial Fluid Pressure and Drug Distribution in Heterogeneous Tumor Nodules during Intraperitoneal Chemotherapy. Drug Deliv..

[41] Zhan W. (2020). Convection Enhanced Delivery of Anti-Angiogenic and Cytotoxic Agents in Combination Therapy against Brain Tumour. Eur. J. Pharm. Sci..

[42] Sweeney P.W., d’Esposito A., Walker-Samuel S., Shipley R.J. (2019). Modelling the Transport of Fluid through Heterogeneous, Whole Tumours in Silico. PLoS Comput. Biol..

[43] Pries A.R., Neuhaus D., Gaehtgens P. (1992). Blood Viscosity in Tube Flow: Dependence on Diameter and Hematocrit. Am. J. Physiol..

[44] Pries A.R., Secomb T.W., Gaehtgens P. (1996). Biophysical Aspects of Blood Flow in the Microvasculature. Cardiovasc. Res..

[45] Pries A.R., Secomb T.W., Tuma R.F., Durán W.N., Ley K. (2008). Blood Flow in Microvascular Networks. Microcirculation.

[46] Pries A.R., Reglin B., Secomb T.W. (2001). Structural Adaptation of Microvascular Networks: Functional Roles of Adaptive Responses. Am. J. Physiol..

[47] Pries A.R., Secomb T.W., Gaehtgens P. (1998). Structural Adaptation and Stability of Microvascular Networks: Theory and Simulations. Am. J. Physiol..

[48] Pries A.R., Cornelissen A.J.M., Sloot A.A., Hinkeldey M., Dreher M.R., Höpfner M., Dewhirst M.W., Secomb T.W. (2009). Structural Adaptation and Heterogeneity of Normal and Tumor Microvascular Networks. PLoS Comput. Biol..

[49] Crawshaw J.R., Flegg J.A., Bernabeu M.O., Osborne J.M. (2023). Mathematical Models of Developmental Vascular Remodelling: A Review. PLoS Comput. Biol..

[50] Salathe E.P., An K.-N. (1976). A Mathematical Analysis of Fluid Movement across Capillary Walls. Microvasc. Res..

[51] Fung Y.C. (1986). Biomechanics-Mechanical Properties of Living Tissues.

[52] Steuperaert M., Labate G.F.D., Debbaut C., De Wever O., Vanhove C. (2017). Mathematical Modeling of Intraperitoneal Drug Delivery: Simulation of Drug Distribution in a Single Tumor Nodule. Drug Deliv..

[53] Kashkooli F.M., Hornsby T.K., Kolios M.C., Tavakkoli J.J. (2023). Ultrasound-Mediated Nano-Sized Drug Delivery Systems for Cancer Treatment: Multi-Scale and Multi-Physics Computational Modeling. WIREs Nanomed. Nanobiotechnol..

[54] Swabb E.A., Wei J., Gullino P.M. (1974). Diffusion and Convection in Normal and Neoplastic Tissues. Cancer Res..

[55] Kashkooli F.M., Soltani M., Rezaeian M., Meaney C., Hamedi M.H., Kohandel M. (2020). Effect of Vascular Normalization on Drug Delivery to Different Stages of Tumor Progression: In-Silico Analysis. J. Drug Deliv. Sci. Technol..

[56] Patlak C.S., Goldstein D.A., Hoffman J.F. (1963). The Flow of Solute and Solvent across a Two-Membrane System. J. Theor. Biol..

[57] Pishko G.L., Astary G.W., Mareci T.H. (2011). Sarntinoranont, Sensitivity Analysis of an Image-Based Solid Tumor Computational Model with Heterogeneous Vasculature and Porosity. Ann. Biomed. Eng..

[58] Gerlowski L.E., Jain R.K. (1986). Microvascular Permeability of Normal and Neoplastic Tissues. Microvasc. Res..

[59] Chou C.-Y., Chang W.-I., Horng T.-L., Lin W.-L. (2017). Numerical Modeling of Nanodrug Distribution in Tumors with Heterogeneous Vasculature. PLoS ONE.

[60] Rippe B., Haraldsson B. (1986). Capillary Permeability in Rat Hindquarters as Determined by Estimations of Capillary Reflection Coefficients. Acta Physiol. Scand..

[61] Shamsi M., Sedaghatkish A., Dejam M., Saghafian M., Mohammadi M., Sanati-Nezhad A. (2018). Magnetically assisted intraperitoneal drug delivery for cancer chemotherapy. Drug Deliv..

[62] Ballard K., Perl W. (1978). Osmotic Reflection Coefficients of Canine Subcutaneous Adipose Tissue Endothelium. Microvasc. Res..

[63] Anderson J.L., Malone D.M. (1974). Mechanism of Osmotic Flow in Porous Membranes. Biophys. J..

[64] Deen W.M. (1987). Hindered Transport of Large Molecules in Liquid-Filled Pores. AICHE J..

[65] Zhao G., Wu J., Xu S., Collins M.W., Long Q., König C.S., Jiang Y., Wang J., Wang J., Padhani A.R. (2007). Numerical Simulation of Blood Flow and Interstitial Fluid Pressure in Solid Tumor Microcirculation Based on Tumor-Induced Angiogenesis. Acta Mech. Sin..

[66] Boucher Y., Baxter L.T., Jain R.K. (1990). Interstitial Pressure Gradients in Tissue-Isolated and Subcutaneous Tumors: Implications for Therapy. Cancer Res..

[67] Soto-Pantoja D.R., Menon J., Gallagher P.E., Tallant E.A. (2009). Angiotensin-(1-7) Inhibits Tumor Angiogenesis in Human Lung Cancer Xenografts with a Reduction in Vascular Endothelial Growth Factor. Mol. Cancer Ther..

[68] Yokoyama Y., Dhanabal M., Griffioen A.W., Sukhatme V.P. (2000). Ramakrishnan, Synergy between Angiostatin and Endostatin: Inhibition of Ovarian Cancer Growth. Cancer Res..

[69] Jain R.K. (2005). Normalization of Tumor Vasculature: An Emerging Concept in Antiangiogenic Therapy. Science.

[70] Boucher Y., Jain R.K. (1992). Microvascular Pressure is the Principal Driving Force for Interstitial Hypertension in Solid Tumors: Implications for Vascular Collapse. Cancer Res..

[71] Tong R.T., Boucher Y., Kozin S.V., Winkler F., Hicklin D.J., Jain R.K. (2004). Vascular Normalization by Vascular Endothelial Growth Factor Receptor 2 Blockade Induces Pressure Gradients across the Vasculature and Improves Drug Penetration in Tumors. Cancer Res..

[72] Kłosowska-Wardęga A., Hasumi, Yoko, Burmakin M., Åhgren A., Stuhr L., Moen I., Reed R.K., Rubin K., Hellberg C. (2009). Combined Anti-Angiogenic Therapy Targeting PDGF and VEGF Receptors Lowers the Interstitial Fluid Pressure in a Murine Experimental Carcinoma. PLoS ONE.

[73] Fan Y., Du W., He B., Fu F., Yuan L., Wu H., Dai W., Zhang H., Wang X., Wang J. (2013). The Reduction of Tumor Interstitial Fluid Pressure by Liposomal Imatinib and its Effect on Combination Therapy with Liposomal Doxorubicin. Biomaterials.

[74] Lee C.-G., Heijn M., Tomaso E.D., Griffon-Etienne G., Ancukiewicz M., Koike C., Park K.R., Ferrara N., Jain R.K., Suit H.D. (2000). Anti-Vascular Endothelial Growth Factor Treatment Augments Tumor Radiation Response under Normoxic or Hypoxic Conditions. Cancer Res..

[75] Deng P.-B., Hu C.-P., Xiong Z., Yang H.-P., Li Y.-Y. (2013). Treatment with EGCG in NSCLC leads to decreasing interstitial fluid pressure and hypoxia to improve chemotherapy efficacy through rebalance of Ang-1 and Ang-2. Chin. J. Nat. Med..

[76] Ferrari F., Sartori M., Milla P., Ronco C., Bellomo R., Kellum J.A., Ricci Z. (2019). Antibiotic Adjustment in Continuous Renal Replacement Therapy. Critical Care Nephrology.

[77] Martin J.D., Fukumura D., Duda D.G., Boucher Y., Jain R.K. (2016). Reengineering the Tumor Microenvironment to Alleviate Hypoxia and Overcome Cancer Heterogeneity. Cold Spring Harb. Perspect. Med..

[78] Stylianopoulos T., Munn L.L., Jain R.K. (2018). Reengineering the Physical Microenvironment of Tumors to Improve Drug Delivery and Efficacy: From Mathematical Modeling to Bench to Bedside. Trends Cancer.

[79] Khawar I.A., Kim J.H., Kuh H.-J. (2015). Improving Drug Delivery to Solid Tumors: Priming the Tumor Microenvironment. J. Control. Release.

[80] Webb T. (2005). Vascular Normalization: Study Examines How Antiangiogenesis Therapies Work. J. Natl. Cancer Inst..

[81] Ho Y.-J., Yeh C.-K. Combination of Anti-Angiogenesis Treatment and Chemotherapy in Solid Tumors by Using Drug-Loaded Nanodroplets Vaporization. Proceedings of the IEEE International Ultrasonics Symposium (IUS).

[82] Park J.-S., Kim I.-K., Han S., Park I., Kim C., Bae J., Oh S.J., Lee S., Kim J.H., Woo D.-C. (2016). Normalization of Tumor Vessels by Tie2 Activation and Ang2 Inhibition Enhances Drug Delivery and Produces a Favorable Tumor Microenvironment. Cancer Cell.

[83] Chauhan V.P., Stylianopoulos T., Martin J.D., Popović Z., Chen O., Kamoun W.S., Bawendi M.G., Fukumura D., Jain R.K. (2012). Normalization of Tomour Blood Vessels Improves the Delivery of Nanomedicines in a Size-Dependent Manner. Nat. Nanotechnol..

[84] Li W., Zhao X., Du B., Li X., Liu S., Yang X.-Y., Ding H., Yang W., Pan F., Wu X. (2016). Gold Nanoparticle–Mediated Targeted Delivery of Recombinant Human Endostatin Normalizes Tumour Vasculature and Improves Cancer Therapy. Sci. Rep..

[85] Birsner A.E., Benny O., D’Amato R.J. (2014). The Corneal Micropocket Assay: A Model of Angiogenesis in the Mouse Eye. J. Vis. Exp..

[86] Muthukkaruppan V.R., Kubai L., Auerbach R. (1982). Tumor-Induced Neovascularization in the Mouse Eye. J. Natl. Cancer Inst..

[87] Lopez E.S., Rizzo M.M., Croxatto J.O., Mazzolini G., Gallo J.E. (2011). Suramab, a Novel Antiangiogenic Agent, Reduces Tumor Growth and Corneal Neovascularization. Cancer Chemother. Pharmacol..

[88] Nicoli S., Presta M. (2007). The Zebrafish/Tumor Xenograft Angiogenesis Assay. Nat. Protoc..

[89] Zhang J., Gao B., Zhang W., Qian Z., Xiang Y. (2018). Monitoring Antiangiogenesis of Bevacizumab in Zebrafish. Drug Des. Dev. Ther..

[90] Kuzmic N., Moore T., Devadas D., Young E.W.K. (2019). Modelling of Endothelial Cell Migration and Angiogenesis in Microfluidic Cell Culture Systems. Biomech. Model. Mechanobiol..

[91] Blache U., Ehrbar M. (2017). Inspired by Nature: Hydrogels as Versatile Tools for Vascular Engineering. Adv. Wound Care.

